# Joint X-ray/neutron structure of *Lentinus similis* AA9_A at room temperature

**DOI:** 10.1107/S2053230X22011335

**Published:** 2023-01-01

**Authors:** Tobias Tandrup, Leila Lo Leggio, Flora Meilleur

**Affiliations:** a Department of Chemistry, University of Copenhagen, Universitetsparken 5, 2100 Copenhagen, Denmark; b Department of Molecular and Structural Biochemistry, North Carolina State University, Campus Box 7622, Raleigh, NC 27695, USA; c Neutron Scattering Division, Oak Ridge National Laboratory, PO Box 2008, Oak Ridge, TN 37831, USA; Universidade Nova de Lisboa, Portugal

**Keywords:** lytic polysaccharide mono­oxygenases, *Lentinus similis* AA9_A, copper metalloenzymes, proton­ation states, neutron crystallography

## Abstract

Lytic polysaccharide monooxygenases (LPMOs) are copper metalloenzymes which cleave polysaccharides oxidatively and are important in pathogen biology, carbon cycling and biotechnology. The *Lentinus similis* family AA9 isoform A (*Ls*AA9_A) has been extensively studied as a model system because its activity towards smaller soluble saccharide substrates has allowed detailed structural characterization of its interaction with a variety of substrates by X-ray crystallography at high resolution. Here, the joint X-ray/neutron room-temperature crystallographic structure of carbohydrate-free *Ls*AA9_A in the copper(II) resting state refined against X-ray and neutron data at 2.1 and 2.8 Å resolution, respectively, is presented. The results provide an experimental determination of the protonation states of the copper(II)-coordinating residues and second-shell residues in *Ls*AA9_A, paving the way for future neutron crystallographic studies of LPMO–carbohydrate complexes.

## Abstract 2

The room-temperature structure of *Lentinus similis* AA9_A was refined jointly against X-ray and neutron crystallographic data extending to 2.1 and 2.8 Å resolution, respectively.

## 1. Introduction

**Figure 1:**
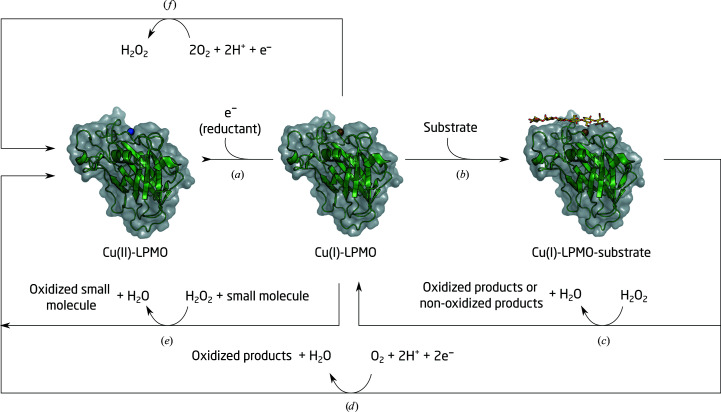
LPMO reaction scheme. Depending on the specific LPMO and the available conditions, one of several reactions may take place. Initial reduction (*a*) and substrate binding (*b*) is followed by the formation of oxidized or non-oxidized chain ends depending on the specfic LPMO, using H_2_O_2_ (*c*) or O_2_ (*d*) as a co-substrate. Oxidizing reactions may also occur in the presence of small molecues (*e*). In the presence of O_2_ but without substrate, the LPMO may produce H_2_O_2_ (*f*).

Copper-containing lytic polysaccharide monooxygenases (LPMOs) are a class of metalloenzymes that have recently been brought to the forefront of research advancing renewable energy and the conversion of biomass to value-added chemicals (Johansen, 2016 ▸), as well as having increasingly recognized biological functions in pathogenesis (Vandhana *et al.*, 2022 ▸). They are classified as auxiliary activities (AA) families AA9–AA11 and AA13–AA17 in the Carbohydrate Active enZymes (CAZy) database (Levasseur *et al.*, 2013 ▸; Drula *et al.*, 2022 ▸). Fungal LPMOs are some of many enzymes secreted by fungi to break down plant matter, namely cellulose, and release glucose. LPMOs randomly oxidize recalcitrant crystalline cellulose, disrupting local crystallinity and creating new cellodextrin chain ends that serve as additional starting points for the endo- and exo-activities of glycoside hydrolases (GHs; Agger *et al.*, 2014 ▸). While LPMOs were first described as monooxygenases, binding molecular dioxygen as a co-substrate, it is now established that LPMOs also have peroxidase/peroxygenase activity (Bissaro *et al.*, 2017 ▸). The monooxygenase reaction requires molecular oxygen, two protons and two single-electron transfers from either small-molecule reductants or electron-donating proteins such as cellobiose dehydrogenases that are co-secreted by fungi (Fig. 1 ▸). Mechanistic details, including the source of protons and the chemical nature of oxygen-activated reaction intermediates, for both the O_2_-based and H_2_O_2_-based mechanisms remain to be elucidated in order to understand how these enzymes carry out carbohydrate oxidation.

Neutron protein crystallography is a powerful tool for investigating protein chemistry because it directly locates H-atom positions in a protein structure (Schröder *et al.*, 2018 ▸). Early X-ray and neutron crystallographic studies of LPMOs from the AA9 family have focused on understanding the activation of O_2_ by *Neurospora crassa* LPMO 9D (*Nc*AA9_D) in the absence of substrate (Bodenheimer *et al.*, 2017 ▸; O’Dell, Swartz *et al.*, 2017 ▸; O’Dell, Agarwal *et al.*, 2017 ▸; Schröder *et al.*, 2021 ▸, 2022 ▸). In addition to the direct determination of protonation states, these structures have revealed the geometry and the chemical nature of the initial Cu—O_2_^−^ and Cu—O_2_H intermediates. These results are important to understand the activation of O_2_ (Fig. 1 ▸ *f*). However, elucidation of the mechanism of carbohydrate oxidation requires the trapping of early intermediates in an LPMO–carbohydrate complex (Fig. 1 ▸ *b*). The insolubility of the substrates of *Nc*AA9_D has so far prevented the investigation of LPMO–O_2_–carbohydrate complexes. Later, *Ls*AA9_A was demonstrated to be active towards soluble oligosaccharides and the first structures of an LPMO enzyme in complex with short soluble carbohydrates were reported (Frandsen *et al.*, 2016 ▸; Simmons *et al.*, 2017 ▸). *Ls*AA9_A has now been used extensively as a model LPMO, including high-resolution crystallo­graphic studies to determine the protonation state of key histidine residues (Banerjee *et al.*, 2022 ▸) and detailed photoreduction studies (Tandrup *et al.*, 2022 ▸). Furthermore, the dependence of *Ls*AA9_A on H_2_O_2_ has been established and a novel twist on the LPMO mechanism has been suggested, at least for this specific enzyme (Brander *et al.*, 2021 ▸). Therefore, *Ls*AA9_A opens the opportunity to analyze substrate-bound enzyme complexes and the activation of H_2_O_2_ using neutron crystallography.

Here, we report the neutron structure of carbohydrate-free *Ls*AA9_A in the copper(II) resting state. The protonation states of catalytic residues at and around the copper center are determined.

## 2. Materials and methods

### 2.1. Protein purification and crystal growth

**Figure 2:**
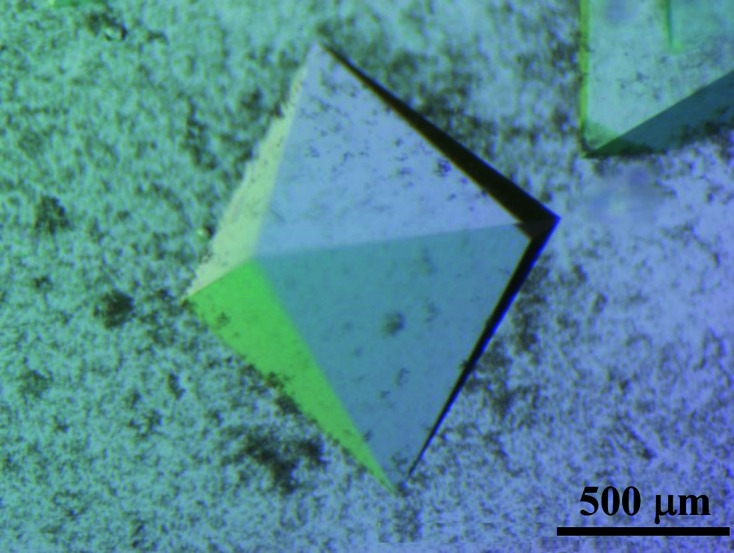
Octahedral crystal of hydrogenated *Ls*AA9_A grown from 100 µl sitting drops.

The fungal enzyme *Ls*AA9_A was expressed in *Aspergillus oryzae*, purified and deglycosylated with endoglycosidase H as described previously (Frandsen *et al.*, 2016 ▸; Simmons *et al.*, 2017 ▸; Tandrup *et al.*, 2020 ▸). The protein sample was pre-incubated with an equimolar amount of copper(II) acetate for 1 h at 4°C prior to crystallization setup. Crystals of *Ls*AA9_A were grown via sitting-drop vapor diffusion by adapting previously reported protocols (Frandsen *et al.*, 2016 ▸). For neutron crystallography, crystals were grown in a nine-well glass-plate and sandwich-box setup (Hampton Research) at 10°C. The sitting drops were equilibrated against 50 ml reservoir solution consisting of 3.0 *M* NaCl, 0.1 *M* citric acid pH 3.5. Large crystals grew from 100 µl sitting drops prepared at a protein concentration ranging from 16 to 20 mg ml^−1^ in 1.3 *M* NaCl, 0.1 *M* citric acid pH 3.5 (Fig. 2 ▸).

### 2.2. Neutron and X-ray data collection at room temperature

Crystals were mounted in thin-walled quartz capillaries (Hampton Research) using hydrogenated crystallization buffer at pH 3.5 from the sandwich box. Excess buffer was removed and plugs of 100 m*M* deuterated citric acid buffer at pD 5.5 (pH electrode reading 5.1) containing 3 *M* NaCl were placed on both sides of the crystal prior to sealing the capillaries to vapor-exchange the crystal solvent water molecules and labile protein H atoms to D_2_O molecules and D atoms, respectively. The pH was increased from 3.5 to 5.5 to prevent disorder of the histidine brace (Frandsen *et al.*, 2017 ▸). The exchange occurred over two weeks prior to neutron data collection.

Neutron time-of-flight diffraction data were collected at room temperature on the MaNDi instrument at the Spallation Neutron Source (Coates & Sullivan, 2020 ▸; Meilleur *et al.*, 2018 ▸). An incident neutron wavelength bandpass of 3–5 Å was used. A total of six diffraction patterns with a Δφ of 10° between frames were collected with an exposure of 48 h per frame. Following neutron diffraction data collection, an X-ray data set was collected from the same crystal at room temperature on a microfocus rotating-anode X-ray diffracto­meter (MicroMax-007 HF, Rigaku). A total of 57 diffraction patterns were collected with a Δφ of 1.0° and an exposure of 45 s per frame.

The neutron data set was reduced using the *Mantid* package (Arnold *et al.*, 2014 ▸) and integrated using three-dimensional profile fitting (Sullivan *et al.*, 2018 ▸). The data were wavelength-normalized using *LAUENORM* from the *LAUEGEN* suite (Helliwell *et al.*, 1989 ▸; Campbell *et al.*, 1998 ▸; Arzt *et al.*, 1996 ▸). The X-ray data were indexed and integrated using *CrysAlis^Pro^* (Rigaku, Woodlands, Texas, USA) and scaled and merged with *AIMLESS* in the *CCP*4 suite (Evans & Murshudov, 2013 ▸; Winn *et al.*, 2011 ▸). Data-collection and processing statistics are summarized in Table 1 ▸. The data and structure have been deposited in the Protein Data Bank (PDB entry 8e1w; Berman *et al.*, 2000 ▸).

### 2.3. Structure refinement

Joint X-ray/neutron refinement was performed using the *Phenix* software suite (Liebschner *et al.*, 2019 ▸) with manual model building performed in *Coot* (Emsley *et al.*, 2010 ▸).

The ligand-free model of *Ls*AA9_A at pH 3.5 (PDB entry 5n04; Frandsen *et al.*, 2017 ▸) stripped of the Cu^2+^ ion and water molecules was used as the starting model. Iterative rounds of refinement and model building were conducted. When the model refined against the X-ray data alone was complete, H and D atoms were generated using the *Phenix ReadySet!* tool as described by Schröder & Meilleur (2020 ▸). The model was further refined against both the X-ray and neutron data sets. Refinement statistics are listed in Table 1 ▸.

## 3. Results and discussion

Neutron and X-ray data sets were collected from the same crystal to 2.8 Å and 2.1 Å resolution, respectively. The *Ls*AA9_A model refined jointly against the X-ray and neutron data includes key LPMO structural features at the active site.

### 3.1. Vapor exchange

**Figure 3:**
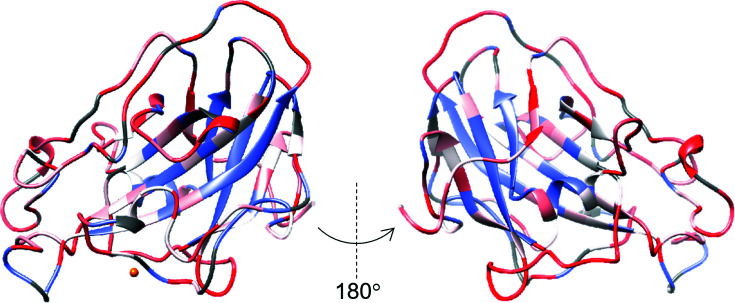
Hydrogen/deuterium exchange of the backbone amide groups. Full (100% D, 0% H) exchange is plotted in red. No exchange (0% D, 100% H) is plotted in blue. Copper(II) is represented by an orange sphere.

Neutron diffraction data collection requires hydrogen to be exchanged for deuterium to increase the signal-to-noise ratio of the data and the visibility of the hydrogen/deuterium positions (Meilleur, 2020 ▸). Vapor exchange was performed over a period of two weeks, which is typical of the time used in a neutron protein crystallography experiment to exchange hydrogen for deuterium (O’Dell *et al.*, 2016 ▸). The hydrogen–deuterium exchange pattern for the backbone amide groups is presented in Fig. 3 ▸. As expected, the outer loops show a high level of exchange, while the inner β-sheets undergo limited exchange. However, the overall exchange is low (35%), indicating that at this low pH longer times are required to achieve higher overall exchange. The pH dependence of the amide hydrogen-exchange rate has previously been examined using mass-spectrometry experiments. These studies suggest that the exchange time required at pH 5.5 to achieve the same level of exchange as at pH 7.5 is 100 times longer (Li *et al.*, 2014 ▸). This time scale is challenging to achieve when planning for neutron data collection.

### 3.2. The histidine brace

As previously described for *Ls*AA9_A, the Cu^2+^ ion in the joint X-ray/neutron structure presented here is coordinated by the N-terminal amino group and N^δ^ of His1, N^ɛ^ of His78 and a water molecule in the equatorial position (H_2_O-eq), while the OH group of Tyr164 and a water molecule are located close to the axial coordination sites. The distances of the copper to the ligands are listed in Table 2 ▸.

The crystals were grown at pH 3.5. At this pH the histidine brace is disordered, with His78 adopting two conformations as reported previously for *Ls*AA9_A (Frandsen *et al.*, 2017 ▸). Ordering of the histidine brace requires the pH to be increased. The pH increase is typically achieved by soaking crystals directly in buffer at pH 5.5. Here, to avoid compromising the diffraction quality of the large crystals, the pH was increased by vapor diffusion.

**Figure 4:**
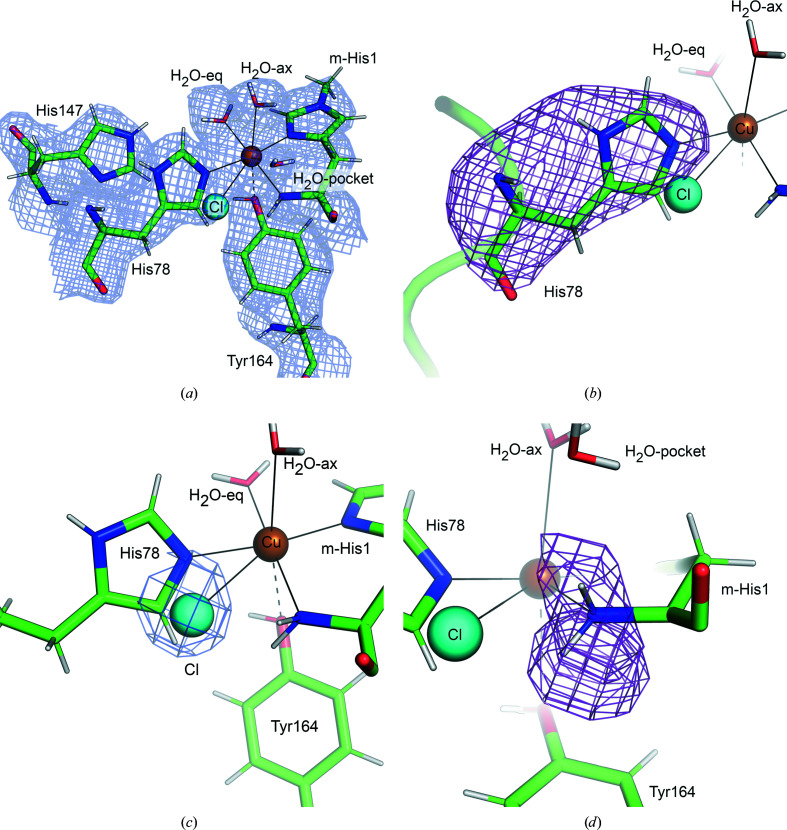
The *Ls*AA9_A active site modeled in the joint X-ray/neutron structure at room temperature. (*a*) 2*F*_o_ − *F*_c_ electron-density map in light blue contoured at 1.0σ. (*b*) *F*_o_ − *F*_c_ neutron scattering-length density omit map for His78 in purple contoured at 3.0σ. His78 is modeled in a single conformation with 75% occupancy. (*c*) *F*_o_ − *F*_c_ electron-density omit map for Cl^−^ in dark blue contoured at 3.0σ. The Cl^−^ ion is modeled with 25% occupancy. (*d*) *F*_o_ − *F*_c_ neutron scattering-length density omit map for the N-terminal amino group in purple contoured at 3.0σ, indicating that the N-terminal amino group is neutral (NH_2_).

Clear electron and neutron scattering-length densities were observed for His78 coordinated to Cu^2+^, confirming that vapor pH exchange drove ordering of the histidine brace (Figs. 4 ▸ *a* and 4 ▸ *b*). No residual *F*_o_ − *F*_c_ electron density was observed for an alternate conformation of His78 flipped away from the active-site Cu^2+^. However, residual *F*_o_ − *F*_c_ electron density indicated that a Cl^−^ ion from the crystallization condition alternatively coordinates the Cu^2+^ ion instead of His78 as observed in the structure of *Ls*AA9_A previously solved at pH 3.5 (Frandsen *et al.*, 2017 ▸; Fig. 4 ▸ *c*). The occupancies of His78 and Cl^−^ refined to 0.75 and 0.25, respectively. The N-terminal amino group is neutral (Fig. 4 ▸ *d*).

### 3.3. Active-site waters

**Figure 5:**
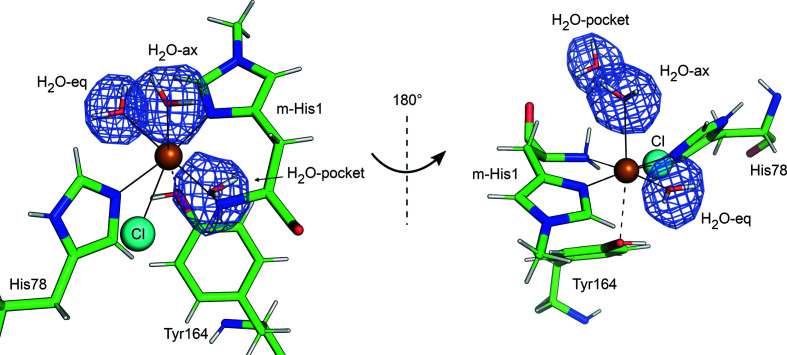
Active-site water molecules. *F*_o_ − *F*_c_ electron-density omit map for H_2_O-eq, H_2_O-ax and H_2_O-pocket in dark blue contoured at 7.0σ.

The active sites of LPMOs are readily prone to X-ray-induced photochemistry, as shown by photoreduction studies on families AA9, AA10 and AA13 (Gudmundsson *et al.*, 2014 ▸; Muderspach *et al.*, 2019 ▸; Banerjee *et al.*, 2022 ▸). At the active site of LPMO, photoreduction causes disorder (and ultimately the disappearance) of the copper-coordinating water molecules in the copper axial and equatorial positions and other geometrical changes. An advantage of neutron diffraction in the structural characterization of metalloenzymes is the lack of radiation-induced in-beam chemistry (Bodenheimer *et al.*, 2017 ▸; Schröder & Meilleur, 2021 ▸). While joint refinement against neutron and X-ray data is a standard approach (O’Dell *et al.*, 2016 ▸), the structure refined against the X-ray data alone must be carefully examined when planning the joint X-ray/neutron refinement of a metalloenzyme. For the joint refinement performed here, we collected an X-ray data set on a home source. The X-ray data show clear *F*_o_ − *F*_c_ electron-density peaks for the copper equatorial and axial water molecules, as well as for the pocket water (Fig. 5 ▸). The distances from H_2_O-eq and H_2_O-ax to Cu^2+^ are 2.1 and 2.7 Å, respectively, confirming that the copper ion is in the resting oxidation state, copper(II), and validating the use of the X-ray data for joint refinement (Tandrup *et al.*, 2022 ▸).

### 3.4. Residues near the active site: Tyr164 and His147

**Figure 6:**
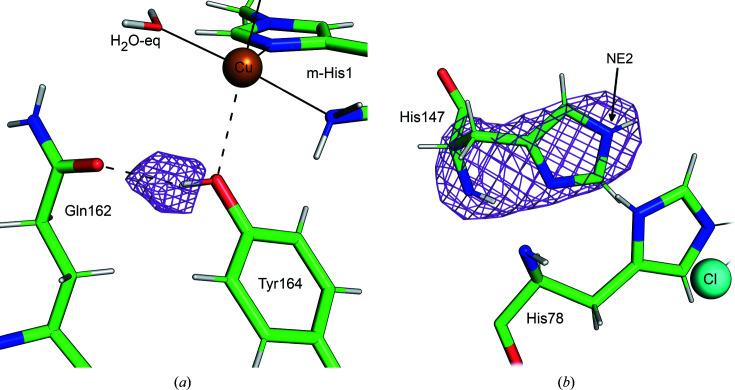
Conserved residues near the copper center. (*a*) *F*_o_ − *F*_c_ neutron scattering-length density omit map for the hydroxy group of Tyr164 in purple contoured at 3.0σ. The hydrogen bond between Tyr164 and Gln162 OE1 is represented by a black dashed line. (*b*) *F*_o_ − *F*_c_ neutron scattering-length density omit map for His147 in purple contoured at 3.0σ, indicating that His147 is singly protonated at the NE2 position.

The LPMO family AA9 active site includes a conserved tyrosine residue. The tyrosine hydroxy group is in the second axial coordination site of the copper, but the Cu–O_Tyr_ distance is too long to form a Cu—O_Tyr_ bond. Recent analysis of X-ray structures at low X-ray dose have confirmed that on binding oligosaccharide the distance to Tyr164 is shortened (Tandrup *et al.*, 2022 ▸). In the structure presented here, Tyr164 is positioned in the axial position of the copper with a Cu–O_Tyr_ distance of 2.80 Å. The Tyr164 OH group forms a hydrogen bond to the side-chain carbonyl group of Gln162 with an O_Tyr164_–O_Gln162_ distance of 2.72 Å. An *F*_o_ − *F*_c_ nuclear map calculated omitting the D atom of the Tyr164 hydroxy group show a clear positive peak at 3.0σ (Fig. 6 ▸ *a*). It will be interesting to observe the effect of saccharide binding on this hydrogen bond in future neutron structures.

His147, a conserved second-shell residue, is singly proton­ated at the NE2 position (Fig. 6 ▸ *b*). This confirms that in this conformation His147 remains neutral at low pH (Schröder *et al.*, 2022 ▸), as also shown by analysis of high-resolution X-ray structures (Banerjee *et al.*, 2022 ▸).

## 4. Conclusion

The protonation state of key amino acids in the active site of *Ls*AA9_A could be determined despite the modest resolution of the neutron data. For future studies, longer pH exchange times are advised, as a small amount of disorder remained at the histidine brace. Perdeuteration allows diffraction from similar-sized crystals to extend to higher resolution by eliminating the incoherent scattering of hydrogen which contributes to the high background. The heterologous expression of *Ls*AA9_A was recently optimized in *Escherichia coli*, which opens the opportunity to perdeuterate the protein (Hernández-Rollán *et al.*, 2021 ▸). *Ls*AA9_A produced in *E. coli* was crystallized and high-quality X-ray structures were obtained, including a complex with cellotriose (Banerjee *et al.*, 2022 ▸; Tandrup *et al.*, 2022 ▸). Future neutron structural investigation of *Ls*AA9_A in complex with carbohydrate ligands will be greatly enhanced by the use of perdeuterated protein.

## Supplementary Material

PDB reference: Neutron crystal structure of *Lentinus similis* AA9_A at room temperature, 8e1w

## Figures and Tables

**Table 1:** Neutron and X-ray data-collection and joint refinement statistics Values in parentheses are for the highest resolution shell.

	X-ray	Neutron
Wavelength (Å)	1.54	3.0–5.0
Resolution range (Å)	30.67–2.10 (2.18–2.10)	14.8–2.80 (2.90–2.80)
Space group	*P*4_1_32	*P*4_1_32
*a*, *b*, *c* (Å)	126.469, 126.469, 126.469	126.469, 126.469, 126.469
α, β, γ (°)	90, 90, 90	90, 90, 90
Total reflections	212841 (21125)	91012 (7436)
Unique reflections	20749 (2019)	8942 (869)
Multiplicity	10.3 (10.5)	10.2 (8.6)
Completeness (%)	99.87 (99.95)	99.3 (99.9)
Mean *I*/σ(*I*)	14.16 (2.09)	10.1 (4.8)
*R*_merge_	0.1758 (1.482)	0.262 (0.290)
*R*_meas_	0.1851 (1.559)	0.275 (0.308)
*R*_p.i.m._	0.0571 (0.4802)	0.081 (0.102)
CC_1/2_	0.988 (0.746)	0.920 (0.348)
Reflections used in refinement	20738	8850
Reflections used for *R*_free_	1033	886
*R*_work_	0.1484	0.2251
*R*_free_	0.1702	0.2598
No. of non-H atoms		
Total	1931	
Macromolecule	1798	
Cu	1	
Cl	1	
*N*-Acetylglucosamine	14	
Solvent	117	
Protein residues	235	
R.m.s.d., bond lengths (Å)	0.015	
R.m.s.d., angles (°)	1.27	
Ramachandran favored (%)	91.81	
Ramachandran allowed (%)	8.19	
Ramachandran outliers (%)	0.00	
Rotamer outliers (%)	1.53	
Clashscore	1.42	
Average *B* factor (Å^2^)		
Overall	33.56	
Macromolecule	32.92	
Cu	35.33	
Cl	33.0	
*N*-Acetylglucosamine	50.54	
Solvent	40.84	

**Table 2:** Distances within the *Ls*AA9_A copper site

Ligand	Copper–ligand distance (Å)
His1 N	2.2
His1 N^δ^	2.0
His78 N^ɛ^ (0.75)/Cl^−^ (0.25)†	2.1/2.5
H_2_O-eq	2.1
H_2_O-ax	2.7
Tyr164 O^η^	2.8

† The occupancies of His78 and the Cl^−^ ligand are shown in parentheses.

## References

[bb1] Agger, J. W., Isaksen, T., Várnai, A., Vidal-Melgosa, S., Willats, W. G. T., Ludwig, R., Horn, S. J., Eijsink, V. G. H. & Westereng, B. (2014). *Proc. Natl Acad. Sci. USA*, **111**, 6287–6292. 10.1073/pnas.1323629111 PMC4035949 24733907

[bb2] Arnold, O., Bilheux, J. C., Borreguero, J. M., Buts, A., Campbell, S. I., Chapon, L., Doucet, M., Draper, N., Ferraz Leal, R., Gigg, M. A., Lynch, V. E., Markvardsen, A., Mikkelson, D. J., Mikkelson, R. L., Miller, R., Palmen, K., Parker, P., Passos, G., Perring, T. G., Peterson, P. F., Ren, S., Reuter, M. A., Savici, A. T., Taylor, J. W., Taylor, R. J., Tolchenov, R., Zhou, W. & Zikovsky, J. (2014). *Nucl. Instrum. Methods Phys. Res. A*, **764**, 156–166.

[bb3] Arzt, S., Campbell, J. W., Hao, Q., Nguti, D., Harding, M. M., Helliwell, J. R., Bradbrook, G., Habash, J., Nieh, Y. P. & Snell, E. H. (1996). *Acta Cryst.* A**52**, C50.

[bb4] Banerjee, S., Muderspach, S. J., Tandrup, T., Frandsen, K. E. H., Singh, R. K., Ipsen, J. O., Hernández-Rollán, C., Nørholm, M. H. H., Bjerrum, M. J., Johansen, K. S. & Lo Leggio, L. (2022). *Biomolecules*, **12**, 194. 10.3390/biom12020194 PMC8961595 35204695

[bb5] Berman, H. M., Westbrook, J., Feng, Z., Gilliland, G., Bhat, T. N., Weissig, H., Shindyalov, I. N. & Bourne, P. E. (2000). *Nucleic Acids Res.* **28**, 235–242. 10.1093/nar/28.1.235 PMC102472 10592235

[bb6] Bissaro, B., Røhr, Å. K., Müller, G., Chylenski, P., Skaugen, M., Forsberg, Z., Horn, S. J., Vaaje-Kolstad, G. & Eijsink, V. G. H. (2017). *Nat. Chem. Biol.* **13**, 1123–1128. 10.1038/nchembio.2470 28846668

[bb7] Bodenheimer, A. M., O’Dell, W. B., Stanley, C. B. & Meilleur, F. (2017). *Carbohydr. Res.* **448**, 200–204. 10.1016/j.carres.2017.03.001 28291519

[bb8] Brander, S., Tokin, R., Ipsen, J. Ø., Jensen, P. E., Hernández-Rollán, C., Nørholm, M. H. H., Lo Leggio, L., Dupree, P. & Johansen, K. S. (2021). *ACS Catal.* **11**, 13848–13859.

[bb9] Campbell, J. W., Hao, Q., Harding, M. M., Nguti, N. D. & Wilkinson, C. (1998). *J. Appl. Cryst.* **31**, 496–502.

[bb10] Coates, L. & Sullivan, B. (2020). *Methods Enzymol.* **634**, 87–99. 10.1016/bs.mie.2019.11.020 32093844

[bb11] Drula, E., Garron, M. L., Dogan, S., Lombard, V., Henrissat, B. & Terrapon, N. (2022). *Nucleic Acids Res.* **50**, D571–D577. 10.1093/nar/gkab1045 PMC8728194 34850161

[bb12] Emsley, P., Lohkamp, B., Scott, W. G. & Cowtan, K. (2010). *Acta Cryst.* D**66**, 486–501. 10.1107/S0907444910007493 PMC2852313 20383002

[bb13] Evans, P. R. & Murshudov, G. N. (2013). *Acta Cryst.* D**69**, 1204–1214. 10.1107/S0907444913000061 PMC3689523 23793146

[bb14] Frandsen, K. E., Simmons, T. J., Dupree, P., Poulsen, J. C., Hemsworth, G. R., Ciano, L., Johnston, E. M., Tovborg, M., Johansen, K. S., von Freiesleben, P., Marmuse, L., Fort, S., Cottaz, S., Driguez, H., Henrissat, B., Lenfant, N., Tuna, F., Baldansuren, A., Davies, G. J., Lo Leggio, L. & Walton, P. H. (2016). *Nat. Chem. Biol.* **12**, 298–303. 10.1038/nchembio.2029 PMC4817220 26928935

[bb15] Frandsen, K. E. H., Poulsen, J. N., Tandrup, T. & Lo Leggio, L. (2017). *Carbohydr. Res.* **448**, 187–190. 10.1016/j.carres.2017.03.010 28364950

[bb16] Gudmundsson, M., Kim, S., Wu, M., Ishida, T., Momeni, M. H., Vaaje-Kolstad, G., Lundberg, D., Royant, A., Ståhlberg, J., Eijsink, V. G. H,, Beckham, G. T. & Sandgren, M. (2014). *J. Biol. Chem.* **289**, 18782–18792. 10.1074/jbc.M114.563494 PMC4081921 24828494

[bb17] Helliwell, J. R., Habash, J., Cruickshank, D. W. J., Harding, M. M., Greenhough, T. J., Campbell, J. W., Clifton, I. J., Elder, M., Machin, P. A., Papiz, M. Z. & Zurek, S. (1989). *J. Appl. Cryst.* **22**, 483–497.

[bb18] Hernández-Rollán, C., Falkenberg, K. B., Rennig, M., Bertelsen, A. B., Ipsen, J. Ø., Brander, S., Daley, D. O., Johansen, K. S. & Nørholm, M. H. H. (2021). *ACS Synth. Biol.* **10**, 897–906. 10.1021/acssynbio.1c00034 33797234

[bb19] Johansen, K. S. (2016). *Biochem. Soc. Trans.* **44**, 143–149. 10.1042/BST20150204 26862199

[bb20] Levasseur, A., Drula, E., Lombard, V., Coutinho, P. M. & Henrissat, B. (2013). *Biotechnol. Biofuels*, **6**, 41. 10.1186/1754-6834-6-41 PMC3620520 23514094

[bb21] Li, J., Rodnin, M. V., Ladokhin, A. S. & Gross, M. L. (2014). *Biochemistry*, **53**, 6849–6856. 10.1021/bi500893y PMC4222528 25290210

[bb22] Liebschner, D., Afonine, P. V., Baker, M. L., Bunkóczi, G., Chen, V. B., Croll, T. I., Hintze, B., Hung, L.-W., Jain, S., McCoy, A. J., Moriarty, N. W., Oeffner, R. D., Poon, B. K., Prisant, M. G., Read, R. J., Richardson, J. S., Richardson, D. C., Sammito, M. D., Sobolev, O. V., Stockwell, D. H., Terwilliger, T. C., Urzhumtsev, A. G., Videau, L. L., Williams, C. J. & Adams, P. D. (2019). *Acta Cryst.* D**75**, 861–877.

[bb23] Meilleur, F. (2020). *Biochemist*, **42**, 16–20.

[bb24] Meilleur, F., Coates, L., Cuneo, M. J., Kovalevsky, A. & Myles, D. A. A. (2018). *Crystals*, **8**, 388.

[bb25] Muderspach, S. J., Tandrup, T., Frandsen, K. E. H., Santoni, G., Poulsen, J.-C. N. & Lo Leggio, L. (2019). *Amylase*, **3**, 41–54.

[bb26] O’Dell, W. B., Agarwal, P. K. & Meilleur, F. (2017). *Angew. Chem. Int. Ed.* **56**, 767–770. 10.1002/anie.201610502 PMC5340418 28004877

[bb27] O’Dell, W. B., Bodenheimer, A. M. & Meilleur, F. (2016). *Arch. Biochem. Biophys.* **602**, 48–60. 10.1016/j.abb.2015.11.033 26592456

[bb28] O’Dell, W. B., Swartz, P. D., Weiss, K. L. & Meilleur, F. (2017). *Acta Cryst.* F**73**, 70–78. 10.1107/S2053230X16020318 PMC5297926 28177316

[bb29] Schröder, G. C. & Meilleur, F. (2020). *J. Vis. Exp.*, e61903. 10.3791/61903 33346193

[bb30] Schröder, G. C. & Meilleur, F. (2021). *Acta Cryst.* D**77**, 1251–1269. 10.1107/S2059798321009025 PMC8489226 34605429

[bb31] Schröder, G. C., O’Dell, W. B., Myles, D. A. A., Kovalevsky, A. & Meilleur, F. (2018). *Acta Cryst.* D**74**, 778–786. 10.1107/S2059798318001626 30082513

[bb32] Schröder, G. C., O’Dell, W. B., Swartz, P. D. & Meilleur, F. (2021). *Acta Cryst.* F**77**, 128–133. 10.1107/S2053230X21002399 PMC8034432 33830078

[bb33] Schröder, G. C., O’Dell, W. B., Webb, S. P., Agarwal, P. K. & Meilleur, F. (2022). *Chem. Sci.* **13**, 13303–13320. 10.1039/d2sc05031e PMC9683017 36507176

[bb34] Simmons, T. J., Frandsen, K. E. H., Ciano, L., Tryfona, T., Lenfant, N., Poulsen, J. C., Wilson, L. F. L., Tandrup, T., Tovborg, M., Schnorr, K., Johansen, K. S., Henrissat, B., Walton, P. H., Lo Leggio, L. & Dupree, P. (2017). *Nat. Commun.* **8**, 1064. 10.1038/s41467-017-01247-3 PMC5651836 29057953

[bb35] Sullivan, B., Archibald, R., Langan, P. S., Dobbek, H., Bommer, M., McFeeters, R. L., Coates, L., Wang, X. P., Gallmeier, F., Carpenter, J. M., Lynch, V. & Langan, P. (2018). *Acta Cryst.* D**74**, 1085–1095. 10.1107/S2059798318013347 PMC6213576 30387767

[bb36] Tandrup, T., Muderspach, S. J., Banerjee, S., Santoni, G., Ipsen, J. Ø., Hernández-Rollán, C., Nørholm, M. H. H., Johansen, K. S., Meilleur, F. & Lo Leggio, L. (2022). *IUCrJ*, **9**, 666–681. 10.1107/S2052252522007175 PMC9438499 36071795

[bb37] Tandrup, T., Tryfona, T., Frandsen, K. E. H., Johansen, K. S., Dupree, P. & Lo Leggio, L. (2020). *Biochemistry*, **59**, 3347–3358. 10.1021/acs.biochem.0c00312 32818374

[bb38] Vandhana, T. M., Reyre, J. L., Sushmaa, D., Berrin, J. G., Bissaro, B. & Madhuprakash, J. (2022). *New Phytol.* **233**, 2380–2396. 10.1111/nph.17921 34918344

[bb39] Winn, M. D., Ballard, C. C., Cowtan, K. D., Dodson, E. J., Emsley, P., Evans, P. R., Keegan, R. M., Krissinel, E. B., Leslie, A. G. W., McCoy, A., McNicholas, S. J., Murshudov, G. N., Pannu, N. S., Potterton, E. A., Powell, H. R., Read, R. J., Vagin, A. & Wilson, K. S. (2011). *Acta Cryst.* D**67**, 235–242. 10.1107/S0907444910045749 PMC3069738 21460441

